# Intraoperative 40 Hz Visual Light Flicker Attenuates Anesthesia/Surgery‐Induced Cognitive Impairments in Elderly Mice With Enhanced Cortical‐Hippocampal Coherence

**DOI:** 10.1002/cns.70809

**Published:** 2026-03-07

**Authors:** Jingyao Jiang, Xin Huang, Jin Liu, Juan Xin, Lanyu Zhang, Zhicheng Yue, Qian Li, Tao Zhu, Peng Liang, Zhiyu Huang, Bhushan Sandeep, Jing Yang, Cheng Zhou

**Affiliations:** ^1^ Department of Anesthesiology, West China Hospital Sichuan University Chengdu China; ^2^ Research Center of Anesthesiology, National‐Local Joint Engineering Research Centre of Translational Medicine of Anesthesiology, West China Hospital Sichuan University Chengdu China; ^3^ Department of Anesthesiology The Third People's Hospital of Chengdu Chengdu Sichuan Province China; ^4^ Department of Cardio‐Thoracic Surgery Chengdu Second People's Hospital Chengdu China

**Keywords:** 40 Hz, electroencephalogram, gamma oscillation, perioperative neurocognitive dysfunction, visual light stimulation

## Abstract

**Background:**

Forty Hz light flicker has shown promise in mitigating cognitive impairments, though its mechanisms remain unclear.

**Aims:**

This study aimed to use perioperative neurocognitive dysfunction (PND) as a unique model of neural damage to provide a broader understanding of the neural mechanisms underlying the cognitive improvements associated with 40 Hz visual stimulation and offer new insights into the clinical application of PND treatment.

**Materials and Methods:**

Postoperative cognitive function was assessed through behavioral tests. Male and female mice received various visual light flicker stimuli, including 40 Hz, random, continuous, or no light. Local field potentials were recorded from the hippocampal dentate gyrus (DG) and primary visual cortex.

**Results:**

Our results show that among the stimuli, only the 40 Hz flicker improved cognitive function, impaired by anesthesia or surgery. Intraoperative 40 Hz stimulation activated the primary visual cortex and was correlated with enhanced gamma coherence between this region and the hippocampal DG, a coherence that surgery itself notably reduced. This preserved functional connectivity. Additionally, hippocampal DG activity was enhanced, particularly in the gamma frequency range.

**Conclusion:**

Our results suggest that 40 Hz flicker mitigates anesthesia/surgery‐induced cognitive deficits, potentially through modulating gamma coherence between the visual cortex and hippocampus. These findings provide insights into PND prevention and the neural mechanisms underlying 40 Hz‐induced cognitive benefits.

## Graphical abstract

Light flicker activated the visual cortex intraoperatively, including: a reduction in electrical activity suppression, and enhancement of high‐frequency gamma wave power. Moreover, its effects extended beyond the visual cortex, reversing the decline in gamma frequency coherence between the visual cortex and hippocampus and maintaining functional connectivity between the hippocampus and cortex. A significant increase in gamma frequency power was observed in the hippocampus.

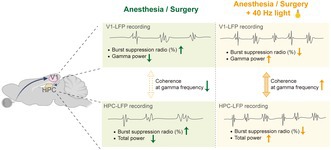

## 1 Introduction

Owing to the unique advantages inherent in sensory stimulation, the evaluation and understanding of its effects on cognitive function improvement have become a prominent area of research in recent years [1, 2]. As is well known, the sensory system is closely linked to cognitive function in the brain [3]. Therefore, multisensory stimulation, such as light, sound, and tactile stimuli, can serve as a non‐pharmacological strategy to improve cognitive dysfunction [4, 5, 6]. Sensory‐based approaches to improving cognitive dysfunction are non‐invasive, have low side effects, and do not require complex equipment or extended training. These approaches can be rapidly implemented using standardized parameters (e.g., light intensity, frequency) and do not rely on active patient participation, making them more widely applicable to a broader patient population than traditional drug or surgical treatments [7, 8, 9, 10].

As one of the commonly used non‐invasive sensory stimulation methods, visual light‐flicker stimulation therapy, using artificial or natural light with varying wavelengths and intensities, has shown promising results in improving cognitive function in the context of general cognitive decline or neurodegenerative diseases, such as Alzheimer's disease (AD) and mild cognitive impairment [5]. Although controversial [11], particularly, recent studies published in authoritative journals have shown that 40 Hz light flicker exerts a greater effect on improving cognitive function compared to other frequencies by enhancing gamma frequency power in brain regions beyond the primary visual cortex, thereby producing therapeutic effects through multiple mechanisms [12, 13, 14, 15, 16]. The therapeutic mechanisms of gamma frequency light flicker primarily involve the reduction of AD‐related pathological proteins, such as amyloid‐beta and phosphorylated tau [12], thereby slowing the progression of neurodegenerative changes, as well as the preservation of neuronal and synaptic density across multiple brain regions [3, 13]. Additionally, it enhances the synchronization and functional connectivity of neural networks and promotes neuroprotection and brain health through the regulation of glial cells [14]. Therefore, the gamma frequency induced by 40 Hz sensory stimulation is a key mechanism in improving cognitive decline in AD models. However, the effects of 40 Hz sensory stimulation have not been validated in other models. Particularly, its efficacy in perioperative contexts remains unexplored [7].

Perioperative neurocognitive dysfunction (PND) is a common complication of the central nervous system observed in elderly patients following anesthesia and surgery [17, 18, 19]. The incidence of PND increases with age, particularly in individuals with underlying cerebral vulnerabilities or those undergoing major surgical procedures [20, 21]. PND encompasses a variety of cognitive impairments, including difficulty concentrating, memory and learning deficits, and executive dysfunction, that generally arise in the postoperative period [22]. While some long‐term follow‐up studies have found no definitive association between PND and subsequent dementia [23], PND is widely viewed as a marker of cerebral vulnerability [24, 25, 26] and is associated with an accelerated trajectory of cognitive decline in older adults [25, 27, 28, 29]. Thus, there is an urgent need for effective strategies to both prevent and treat PND [21, 30, 31]. Given that PND is associated with long‐term cognitive decline, it suggests that sensory stimulation may also benefit cognitive decline in patients with PND. However, no research has yet explored the use of 40 Hz light‐flicker stimulation to improve cognitive function in this context.

Therefore, this study aimed to use PND as a unique model of neural damage to provide a broader understanding of the neural mechanisms underlying the cognitive improvements induced by 40 Hz stimulation and offer new insights into the clinical application of PND treatment. The study explores the impact of visual light flicker on PND and its underlying neural mechanisms, particularly whether it can exert effects through the visual cortex and neural circuits beyond the visual cortex, with a focus on the synchronization of activity within the visual cortex–hippocampal circuit.

## 2 Methods and Materials

### 2.1 Animals

Both age‐matched male (*n* = 14) and female (*n* = 8) C57BL/6J mice (14–16 months, 28–35 g) were obtained from Chengdu Dossy Experimental Animals Co. Ltd. (China) and housed under standard conditions: a 12‐h light/dark cycle, ad libitum food and water, 20%–50% humidity, and a temperature of 21°C–25°C.

### 2.2 Anesthesia and Surgery Procedure

A surgery‐induced cognitive impairment model was created via unilateral nephrectomy [32]. Mice were anesthetized with 3% isoflurane for induction and 1.5% isoflurane in 100% O_2_ for maintenance (1 L/min). The surgery, involving a midline incision and left kidney removal, lasted 20 min with 2 h of anesthesia [33]. Afterward, 3 mg/kg bupivacaine was administered via local infiltration into the surgical incision site, and mice were allowed to recover for 30 min before returning to their cages. Control mice received no anesthesia or surgery.

### 2.3 Experimental Settings

Two main experiments were conducted to investigate the potential protective effects of visual light stimulation on cognitive function and to determine the optimal visual light treatment for PND. In the first experiment, mice were randomly assigned into five groups: control, anesthesia/surgery (A/S), A/S + 40 Hz, A/S + random frequency light (A/S + RL), and A/S + long bright light (A/S + LL), following established light treatment protocols in mice [12].

In the second experiment, the goal was to assess the effect of the optimal visual light treatment on electrophysiological measures, including spectral power and coherence across different frequency bands. A separate cohort of mice with chronic electrode implants was used for electrophysiological recordings to prevent the confounding effects of multiple surgeries and anesthetic events on behavioral outcomes. Control, A/S, and A/S + 40 Hz groups were included in this experiment. All groups underwent the same duration of anesthesia and surgery, during which 1‐h EEG recordings were performed. Concurrently with the EEG recording, the A/S + 40 Hz group received 40 Hz visual light stimulation.

Considering the potential influence of sex differences, the initial experiments utilized male mice exclusively. Following editorial recommendations, data from female mice were subsequently incorporated. Results are presented separately by sex owing to differences in experimental batches.

### 2.4 Visual Light Stimulation Protocol

Mice were positioned in a dark chamber with an LED bulb fixed between their eyes, and their eyelids were gently held open with hypoallergenic ophthalmological tape to ensure consistent light delivery throughout the stimulation. The visual light stimulation commenced at the onset of the surgical procedure and was maintained for 1 h, encompassing both the surgery and the subsequent anesthesia period. During this period, mice were exposed to one of three light conditions: 40 Hz (regular 12.5 ms pulses), random frequency (1–80 Hz random intervals, 40 Hz mean, matched pulse width and total photon flux), or prolonged bright light (continuous illumination, 0 Hz). To ensure consistent delivery of the light stimulus and to prevent variable attenuation due to spontaneous eyelid closure, the mice's eyelids were gently held open with medical‐grade ophthalmological tape for the duration of the light stimulation. To minimize retinal damage, the flicker parameters were set to a color temperature of 3000 ± 500 K and an illumination intensity of 250–750 lx, as supported by safety studies in rodents [8] and their application in mouse models [34].

### 2.5 Behavioral Assessments

#### 2.5.1 Barnes Maze (BM)

Mice underwent the BM test on Days 1, 3, 7, and 14 post‐surgery to assess spatial learning and memory [35]. The maze consisted of a circular platform with multiple holes around its periphery. One hole led to a fixed dark target chamber, the location of which remained constant for each mouse throughout all trials and testing days. During spatial acquisition training (conducted over 4 consecutive days with 3 trials per day), each trial proceeded as follows: a mouse was placed in the center of the platform and allowed a maximum of 180 s to locate and enter the target hole. If the mouse failed to find the target within this period, it was gently guided to it by the experimenter. Upon entering the target chamber (either independently or after guidance), the mouse remained there for 60 s before being returned to its home cage. The primary behavioral metric was the latency to first enter the target hole. For animals that did not find the target within 180 s, the latency was recorded as 180 s for analysis.

#### 2.5.2 Open Field Test (OFT)

The OFT was performed on the 1st day following surgery. Mice were placed into an open field box (50 cm × 50 cm × 40 cm) for 5 min. The Super‐maze behavioral tracking software was used to record: the total distance traveled, and the time spent in the center, border, and corner areas.

#### 2.5.3 Novel Object Recognition Test (NOR)

One day following the OFT, the NOR test was administered to the mice in a square arena (50 cm × 50 cm × 40 cm). Two identical objects (e.g., cubes or pyramids, approximately 4 cm in each dimension) were placed in the arena, each positioned 10 cm away from the side walls. The roles of the two identical objects as “familiar” or “novel” were systematically counterbalanced across animals within each experimental group. The objects were made of inert materials (e.g., plastic), had simple geometric shapes, and were firmly fixed to the floor to prevent displacement. Both objects presented a smooth surface with no inherent features that would allow climbing. Mice were allowed 10 min to explore two identical objects. Following a 1‐h inter‐session interval (mice removed to home cages), one object was replaced with a novel object, and mice were returned to the arena for an additional 10 min of exploration. Exploration was strictly defined as the mouse directing its nose toward and within ~2 cm of the object. Sniffing, touching, or closely investigating the object with vibrissae was counted; sitting on or turning around the object without directed attention was not. Exploration times were recorded, and the exploration ratio was calculated as the time spent on the novel object divided by total exploration time. Additionally, the exploration ratio (Novel/Familiar) was calculated as a secondary measure.

#### 2.5.4 Fear Conditioning Test (FC)

After the NOR test, a FC test was carried out to assess the associative memory of the mice, as detailed in previous study [35]. Mice were placed in a test chamber, conditioned with 3 tone‐foot shock pairings (tone: 2000 Hz, 85 dB, 30 s; shock: 0.7 mA, 2 s) with 1‐min intervals. Twenty‐four hours later, contextual fear memory was tested by returning the mice to the same chamber for 6 min without any stimulus. Two hours later, to assess cued fear memory, the mice were placed in a novel, modified chamber (with distinct visual cues) for a 3‐min baseline exploration, followed by a 3‐min tone exposure (the same tone as during conditioning). Freezing behavior was recorded by an observer blind to the group assignment.

### 2.6 In Vivo Electrophysiology

*Surgery*. Mice were anesthetized with isoflurane, positioned prone, and stabilized using a stereotaxic instrument. Electrodes were implanted in the hippocampus (AP: −2.1 mm, ML: +1.3 mm, DV: −1.7 mm) and primary visual cortex (V1, AP: −2.8 mm, ML: +2.5 mm, DV: −1.5 mm). Four electrodes were implanted: two insulated silver wires (100 μm diameter) with exposed tips for recording local field potentials (LFP) from the target regions, and two stainless steel screws (1 mm diameter) serving as the reference and ground electrodes, respectively, fixed to the skull over the cerebellum. To later verify placement, the recording electrode tips were coated with the fluorescent dye Dil (1,1′‐dioctadecyl‐3,3,3′,3′‐tetramethylindocarbocyanine perchlorate) prior to implantation.

*Recording*. EEG signals were recorded at 1000 Hz using the Pinnacle EEG System.

*Histological Verification*. Within 1 week after the final recording session, mice were transcardially perfused with ice‐cold phosphate‐buffered saline (PBS) followed by 4% paraformaldehyde in PBS. Brains were post‐fixed in the same fixative at 4°C for 24 h and cryoprotected in 30% sucrose‐PBS at 4°C for 48–72 h. The placement of recording electrodes was confirmed by visualizing the pre‐coated Dil dye on coronal brain sections under a fluorescence microscope.

*Analysis*. Continuous EEG/LFP signals were recorded throughout the 1 h stimulation period. A stable 2‐min epoch from the final 10 min was selected for quantitative analysis in MATLAB (ver. 2016b), as it represents a period of stable physiological and anesthetic depth, providing a reliable measure of the sustained intervention effects. Power spectra were calculated using FFT with multi‐taper, focusing on delta (1–5 Hz), theta (5–10 Hz), alpha (10–15 Hz), beta (15–25 Hz), and gamma (25–40 Hz) bands. Coherence and coherogram were computed with tapers = [3 5], winstep = 1, and winsize = 2. Suppression was defined as amplitude < 25 μV, a conventional threshold for identifying periods of low electrocortical activity in rodents. The burst suppression ratio (BSR) was calculated as (Duration of Suppression/Total Duration) × 100%.

### 2.7 Statistical Analyses

All analyses were performed using GraphPad Prism 8.0 (GraphPad Software, San Diego, CA, USA). Results in the text and figures are expressed as mean ± SD. The normality of all data distributions was assessed using the Shapiro–Wilk test, which is appropriate for our sample sizes (*n* < 50). Homogeneity of variances was verified using both the Brown–Forsythe and Bartlett tests. All datasets met the assumptions of normality and homogeneity of variances; therefore, parametric tests were applied. One‐way ANOVA (with Tukey's post hoc test) or unpaired *t*‐test was used to compare data when two or more independent groups were studied. Two‐way ANOVA was used for comparisons when two factors were considered. Statistical significance was set at *p* = 0.05.

## 3 Results

### 3.1 Anesthesia/Surgery Impairs Learning and Memory in Elderly Mice

**FIGURE 1:**
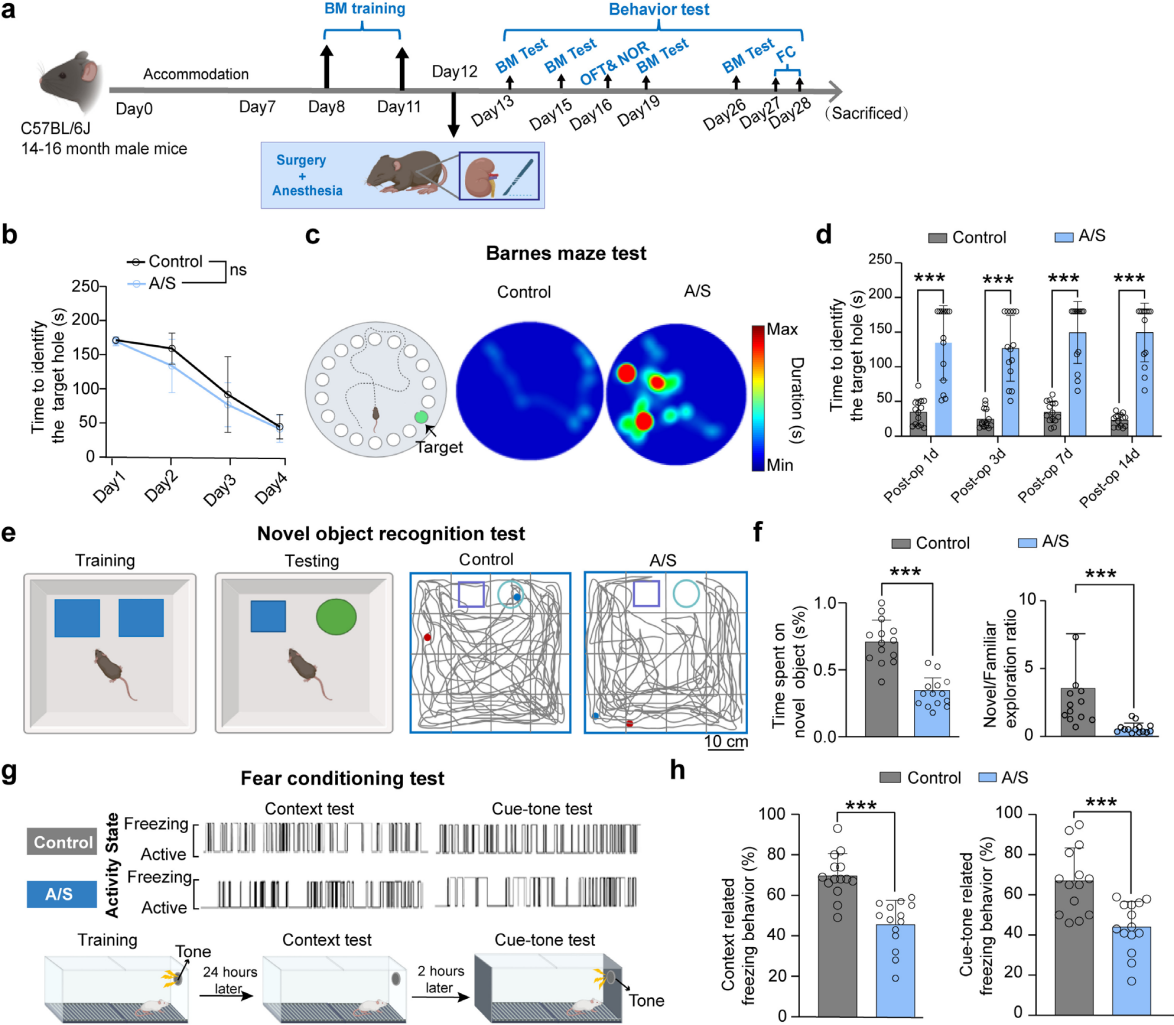
Anesthesia and surgery impaired learning and memory in elderly mice. Mice were randomly assigned to two groups: the control group (Control) and the anesthesia and surgery group (A/S). (a) Timeline of behavioral experiments. Accommodation: Mice were acclimated to the housing environment for 7 days before surgery. (b) Statistical charts showing the time to identify the target hole during training sessions of the Barnes Maze test for male mice. Data are presented as mean ± SD (three trials per day). (c) Representative heat maps depicting the time spent by individual mice in the Barnes maze test. Warmer colors indicate longer residence time. (d) Statistical charts showing the time to identify the target hole on postoperative Days 1, 3, 7, and 14 in the Barnes Maze test. (e) Description of the Novel Object Recognition Test and representative movement trace diagrams; the red and blue dots indicate the start and end points of the trajectory, respectively. (f) Novel Object Recognition Test results. Statistical charts for time spent exploring the novel object as a percentage of total object exploration time (left) and Novel/Familiar exploration ratio (right). (g) Representative movement trace diagrams in the fear conditioning test. (h) Statistical charts showing context‐related freezing behavior (left) and cue‐tone related freezing behavior (right) in the Fear Conditioning Test. Data are presented as mean ± SD with individual data points for each animal (*n* = 14). Results were analyzed using one‐way or two‐way measures ANOVA and *t*‐tests. ***p < 0.001.

The experimental paradigm is illustrated in Figure 1a. As the training sessions progressed, both groups of male mice showed reduced time in locating the target hole (Figure 1b). Notably, surgical intervention significantly impacted the performance of mice during the test sessions (Figure 1c,d). The NOR test showed reduced novel object exploration (Figure 1f, left) and Novel/Familiar exploration ratio (Figure 1f, right) in mice of the A/S group compared to control and A/S + 40 Hz groups. The total exploration time during the NOR test did not differ significantly among groups (Figure S1a), indicating that the reduced novel object exploration in the A/S group was not due to a general lack of locomotor activity. In the open field test (OFT), no significant differences in total distance, time spent in center, border, or corner areas were observed (Figure S1b,c), indicating similar locomotor activity across groups. Lastly, the FC test was used to assess learning and memory in response to aversive stimuli. Methodology and representative movement traces for the FC test are shown in Figure 1g. Mice in the A/S group exhibited significantly shorter freezing behavior in both the context (Figure 1h, left) and tone‐related tests (Figure 1h, right), compared to the control group. These findings collectively suggest that anesthesia and surgery impair multiple domains of learning and memory in elderly mice.

### 3.2 Intraoperative 40 Hz Visual Light Flicker Reduces Cognitive Dysfunction After Anesthesia and Surgery

**FIGURE 2:**
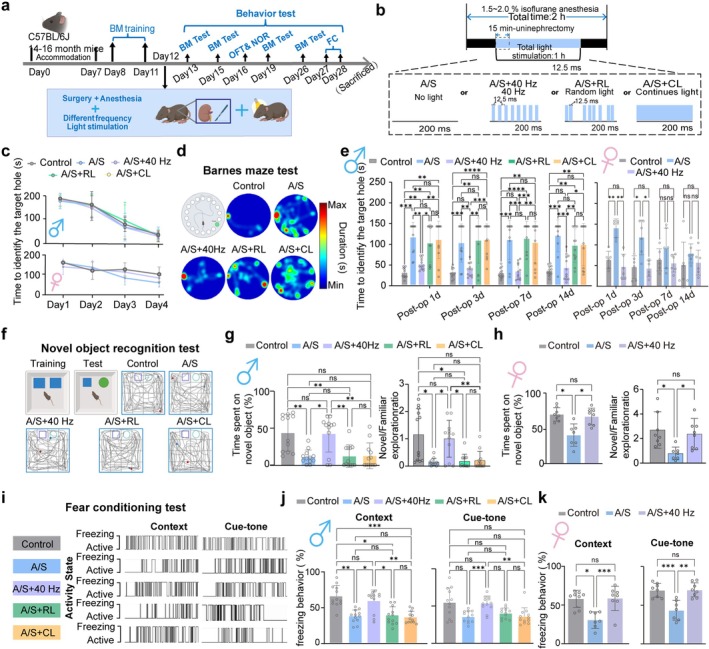
Intraoperative administration of 40 Hz visual light flicker for 1 h mitigates cognitive dysfunction following anesthesia and surgery. Male mice were randomly assigned to five groups: the control group (Control), the anesthesia and surgery group (A/S), the anesthesia and surgery group with 40 Hz visual light flicker (A/S + 40 Hz), the anesthesia and surgery group with random frequency light flicker (A/S + RL), and the anesthesia and surgery group with continuous light flicker (A/S + CL). Female mice were randomly assigned to three groups: Control, A/S, and A/S + 40 Hz. (a) Timeline of behavioral experiments. Accommodation: Mice were acclimated to the housing environment for 7 days before surgery. (b) Top: Experimental protocol for surgery and isoflurane anesthesia with light flicker. Bottom (from left to right): Parametric protocols for absence of light (1), 40 Hz visual light flicker (2), random frequency light flicker (3), and continuous light exposure (4). Light stimulus parameters: 380–750 nm, 500 K ±2600, 250–750 lx. (c) Statistical charts showing the time to identify the target hole during training sessions of the Barnes Maze test for male (UP) and female (Down) mice. (d) Representative heat maps depicting the time spent by individual mice in the Barnes maze test. Warmer colors indicate longer residence time. (e) Statistical charts showing the time to identify the target hole on postoperative days 1, 3, 7, and 14 in the Barnes Maze test for male mice (left) and female mice (right). (f) Description of the Novel Object Recognition Test and representative movement trace diagrams; the red and blue dots indicate the start and end points of the trajectory, respectively. (g) Statistical charts showing the time spent on the novel object (left) and Novel/Familiar exploration ratio (right) in the Open Field Test for male mice. (h) Statistical charts showing the time spent on the novel object (left) and Novel/Familiar exploration ratio (right) in the Open Field Test for female mice. (i) Representative movement trace diagrams in the Fear Conditioning Test. (j) Statistical charts showing context‐related freezing behavior (left) and cue‐tone freezing behavior (right) in the Fear Conditioning Test for male mice. (k) Statistical charts showing context‐related freezing behavior (left) and cue‐tone freezing behavior (right) in the Fear Conditioning Test for female mice. Data are presented as mean ± SD with individual data points for each animal (male: *N* = 12; female: *N* = 8). Results were analyzed using one‐way or two‐way measures ANOVA. *p < 0.05, **p < 0.01, ***p < 0.001.

We examined the effects of light stimuli on PND amelioration. The schematic of the experimental paradigm is presented in Figure 2a. Parametric details of light flicker are illustrated in Figure 2b. During the pre‐surgical training phase, as training sessions increased, all groups of mice showed a reduced time to locate the target hole compared to their initial attempts on the first day (Figure 2c up for males; Figure 2c bottom for females). Postoperatively, compared with the Control and A/S + 40 Hz groups, male mice in the A/S, A/S + RL, and A/S + CL groups took longer to identify the target hole (Figure 2d,e, left). In female mice, a similar impairment in the A/S group was observed, specifically at 1 and 3 days after surgery (Figure 2e, right). The NOR test showed reduced novel object exploration (Figure 2g left for males; Figure 2h left for females) and Novel/Familiar exploration ratio (Figure 2g right for males; Figure 2h right for females) in male mice (A/S, A/S + RL, A/S + CL groups) and female mice (A/S group) compared to control and A/S + 40 Hz groups. The total exploration time during the NOR test did not differ significantly among groups (Figure S1). In the OFT, no significant differences in distance or time spent in different areas were observed (Figure S1d for males; Figure S1e for females). In the FC test, representative movement traces for the FC test are shown in Figure 1i. Male mice in A/S, A/S + RL, and A/S + CL groups exhibited significantly reduced freezing behavior compared with control and A/S + 40 Hz groups in both context and tone tests (Figure 2j, left and right respectively). Female mice in the A/S group showed similarly reduced freezing compared with control and A/S + 40 Hz groups in both tests (Figure 2k, left and right). The results show that 40 Hz light reduces cognitive dysfunction compared to no light, random‐frequency light, or continuous bright light.

### 3.3 Intraoperative 40 Hz Visual Light Flicker Minimizes Local Field Potential Suppression in the Primary Visual Cortex

**FIGURE 3:**
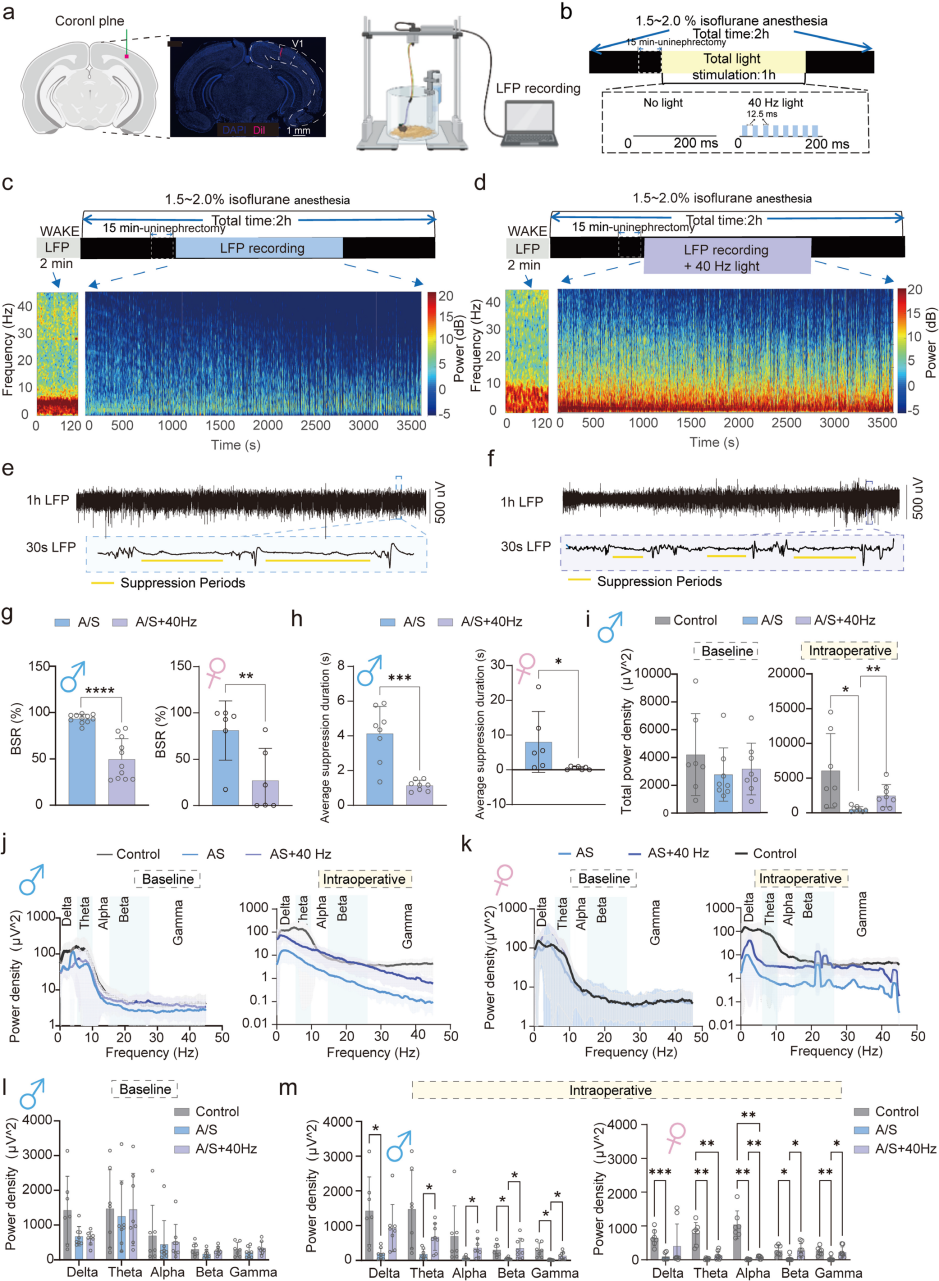
Administration of a 40 Hz visual light‐flicker intraoperatively minimizes local field potential suppression in the primary visual cortex of mice. Male and female mice were randomly allocated to three groups, namely, the control group (control), anesthesia and surgery group (A/S), and anesthesia and surgery administered with 40 Hz visual light‐flicker group (A/S + 40 Hz). (a) Schematic of the recording site in a coronal brain section (created with BioRender; left). The red dot indicates the tip location marked by red fluorescent dye. Photomicrograph showing the electrode track (middle) and schematic of the LFP recording setup (right). Scale bar, 200 μm. (b) Top: Experimental protocol for surgery with light flicker. Bottom: Parametric protocols for absence of light (left) and 40 Hz visual light flicker (right). (c) Top: Timeline of surgery and LFP Recording. Bottom: Continuous A/S group preoperative (left) and intraoperative LFP spectrograms (right). (d) Top: Timeline of surgery and LFP Recording with 40 Hz visual light flicker. Bottom: Continuous A/S + 40 Hz group preoperative and intraoperative LFP spectrograms. (e) Top: Continuous LFP Trace of A/S group for 1 Hour. Bottom: Representative 30‐Second LFP Trace of A/S group. (f) Top: Continuous LFP Trace of A/S + 40 Hz group for 1 h. Bottom: Representative 30‐Second LFP trace of A/S + 40 Hz group. (g) Statistical charts of the BSR of LFP in intraoperative recordings for male (left) and female (right) mice. BSR (%) = (Duration of Suppression Periods/Total Duration of EEG Recording) * 100. (h) Statistical charts of average suppression duration of LFP in intraoperative recordings for male (left) and female (right) mice. (i) Statistical charts of total power density at baseline (left) and during intraoperative periods (right) for male mice. (j) Quantification of power spectral density at baseline (left) and during intraoperative periods (right) for male mice. (k) Quantification of power spectral density at baseline (left) and during intraoperative periods (right) for female mice. (l) Statistical charts of power density across different frequency bands at baseline for male mice. (m) Statistical charts of power density across different frequency bands during intraoperative periods for male mice (left) and female mice (right). Data are presented as mean ± SD with the presentation of data of each individual animal (male: *N* = 8; female: *N* = 8). Results were analyzed by one‐way or two‐way measures ANOVA and *t*‐test. *p < 0.05, **p < 0.01, ***p < 0.001, ****p < 0.0001. BSR, burst suppression ratio; LFP, local field potential; V1, primary visual cortex.

40 Hz visual light flicker is processed by the eyes and relayed to the visual cortex [36, 37]. Anesthesia and surgery induce inhibitory and inflammatory responses that impair visual cortical activity. To assess the effect of 40 Hz flicker on the visual cortex, we recorded LFP from the primary visual cortex in Control, A/S, and A/S + 40 Hz groups. The surgical procedure and electrode setup are shown in Figure 3a. Field potential electrodes were implanted in the primary visual cortex, and the light‐flicker protocol is shown in Figure 3b. Power spectra in Figure 3c,d show higher power in the A/S + 40 Hz group across all frequencies. Raw data for 1 h are shown in Figure 3e,f, with a zoomed‐in 30‐s segment from the last 10 min. LFP suppression (amplitude < ±25 μV) was reduced in the A/S + 40 Hz group, a direct electrophysiological correlate of profound brain inactivation. To assess the depth of cortical suppression under anesthesia, we analyzed the burst suppression ratio (BSR), a quantitative EEG measure of brain inactivation and average suppression duration. Both the burst suppression ratio (BSR) and the average LFP suppression duration were significantly higher in the A/S group than in the A/S + 40 Hz group (Figure 3g,h, left and right for male and female mice respectively). We then compared the overall power of the field potentials. No significant differences were observed in the overall field potential power among the three groups at baseline (Figure 3i, left for male and Figure S2a, left for female). However, the field potential power in the A/S group intraoperatively was significantly lower than that in the other groups (Figure 3i, right for male and Figure S2a, right for female). These findings suggest that 40 Hz visual light flicker during surgery reduces the suppression of brain electrical activity. Under baseline conditions, no significant differences in field potential power across the analyzed frequency bands (delta, theta, alpha, beta, gamma) were observed among all groups in both sexes (Figure 3j, left and l for male and Figure 3k, left and Figure S2b for female). In contrast to the A/S group, the A/S + 40 Hz group showed increased power across nearly all frequency bands, with notable increases in gamma bands—a rhythm implicated in cognitive functions and directly relevant to our 40 Hz stimulation paradigm—in mice of both sexes (Figure 3j, right and Figure 3m left for male; Figure 3k right and Figure 3m right for female). Interestingly, sex‐specific spectral signatures emerged: the increase was particularly pronounced in the alpha band for females, but in the delta band for males.

Overall, 40 Hz visual light flicker alleviates brain activity inhibition in the visual cortex caused by anesthesia and surgery.

### 3.4 Intraoperative 40 Hz Visual Light Flicker Reverses the Reduction in LFP Coherence Between the Primary Visual Cortex and DG at Gamma Frequency

**FIGURE 4:**
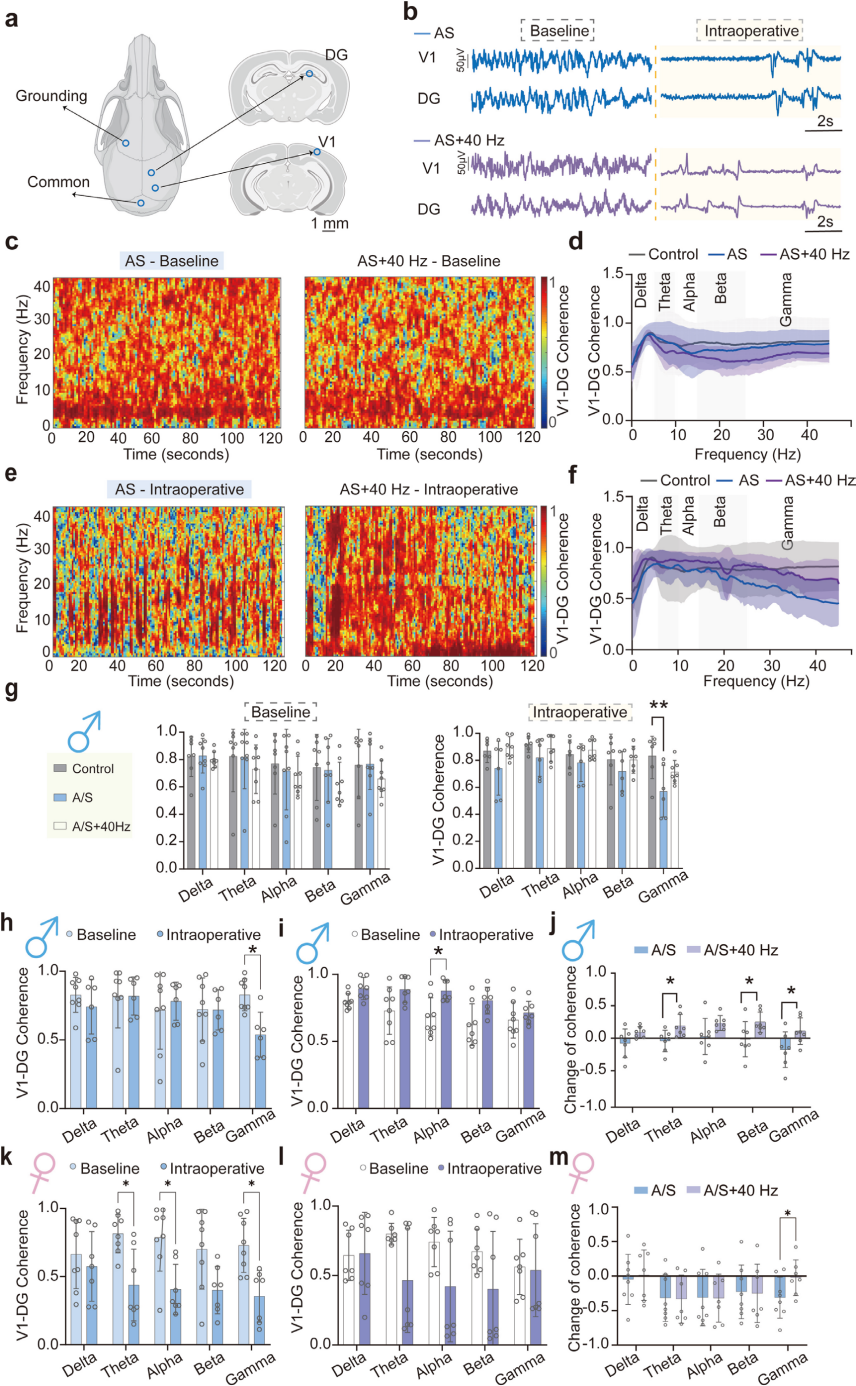
Intraoperative administration of a 40 Hz visual light flicker reversed the reduction of LFP coherence between primary visual cortex and DG at gamma frequency. Male and female mice were randomly allocated to three groups, namely, the control group (control), anesthesia and surgery group (A/S), and anesthesia and surgery administered with 40 Hz visual light‐flicker group (A/S + 40 Hz). (a) Schematic of electrode implantation location (created by biorender). (b) Representative 10‐s LFP Trace in the DG and the V1 of A/S group (top) and A/S + 40 Hz group (bottom) groups at baseline (left) and during intraoperative periods (right). (c) Representative preoperative coherogram of LFP from the V1 to DG of A/S group (left) and A/S + 40 Hz (right) group at baseline. (0 for no correlation, with a maximum value of 1 for perfect correlation). (d) Quantification of coherence of LFP from the V1 to DG at baseline. (e) Representative preoperative coherogram of LFP from the V1 to DG of A/S group (left) and A/S + 40 Hz group (right) during intraoperative periods. (f) Quantification of coherence of LFP from the V1 to DG during intraoperative periods. (g) Statistical charts of coherence statistical charts at baseline (left) and during the intraoperative period (right) across the Control, A/S and A/S + 40 Hz groups for male mice. (h) Quantification of the coherence between the baseline and intraoperative periods in the A/S group for male mice. (i) Quantification of the coherence between the baseline and intraoperative periods in the A/S + 40 Hz group for male mice. (j) Quantification of coherence difference between baseline and intraoperative periods across the A/S and A/S + 40 Hz groups for male mice. (k) Quantification of the coherence between the baseline and intraoperative periods in the A/S group for female mice. Quantification of the coherence between the baseline and intraoperative periods in the A/S + 40 Hz group for female mice. (m) Quantification of coherence difference between baseline and intraoperative periods across the A/S and A/S + 40 Hz groups for female mice. Data are presented as mean ± SD with the presentation of data of each individual animal (male: *N* = 8; female: *N* = 8). Results were analyzed by one‐way or two‐way ANOVA and *t*‐test. *p < 0.05, **p < 0.01, ***p < 0.001. V1, primary visual cortex; DG, dentate gyrus; LFP, local field potential.

The visual cortex exhibits functional and anatomical connectivity with other areas of the brain [38]. Therefore, 40 Hz visual light stimulation cannot induce isolated activation in the visual cortex, and cognitive activity is generally accompanied by an increase in inter‐regional synchrony of brain activity. We examined changes in functional connectivity from the visual cortex to cognitive brain areas by recording field potentials from the hippocampus and visual cortex, as shown in Figure 4a.

Figure 4b shows typical 10‐s raw signals recorded at baseline and during surgery. Figure 4c,d display coherence spectra for the A/S and A/S + 40 Hz groups at baseline, with no significant differences. Figure 4e shows coherence during anesthesia, and Figure 4f presents coherence for each frequency. In frequencies > 20 Hz, although the A/S group showed a decreasing trend, no significant difference was observed. Statistical analysis revealed no significant differences in coherence across frequency bands at baseline (Figure 4g, left for male and Figure S2c, left for female). However, during surgery, the gamma frequency coherence in the A/S group of male mice significantly decreased (Figure 4g, right). A similar pattern of decreased coherence during surgery was observed in female mice across multiple frequency bands, including theta, alpha, beta, and gamma (Figure S2c, right). A/S + 40 Hz group exhibited an increasing trend compared with the A/S group, though the difference did not reach statistical significance, while alpha coherence increased significantly. Compared with baseline, male and female mice in the A/S group exhibited significantly decreased gamma coherence during anesthesia and surgery (Figure 4h for male and Figure 4k for female). In contrast, coherence was maintained in the A/S + 40 Hz group during these procedures (Figure 4i for male and Figure 4l for female). Further analysis revealed that the increase in coherence from baseline in the high‐frequency beta/gamma bands was significantly greater in the A/S + 40 Hz group relative to the A/S group (Figure 4j for males; Figure 4m for females).

This suggests that the application of 40 Hz visual light flicker during anesthesia and surgery alleviates the reduction in brain functional connectivity at gamma frequency.

### 3.5 Intraoperative 40 Hz Visual Light Flicker Minimizes Local Field Potential Suppression in the DG of Mice

The hippocampus is key for learning and memory [39, 40], and its function is tied to PND [41, 42]. To explore how the activation of the visual cortex during surgery, via enhanced functional connectivity, influences hippocampal activity, we implanted LFP electrodes in the dentate gyrus (DG) of the hippocampus to assess the effect of 40 Hz light‐flicker stimulation on brain electrical activity during anesthesia and surgery.

**FIGURE 5:**
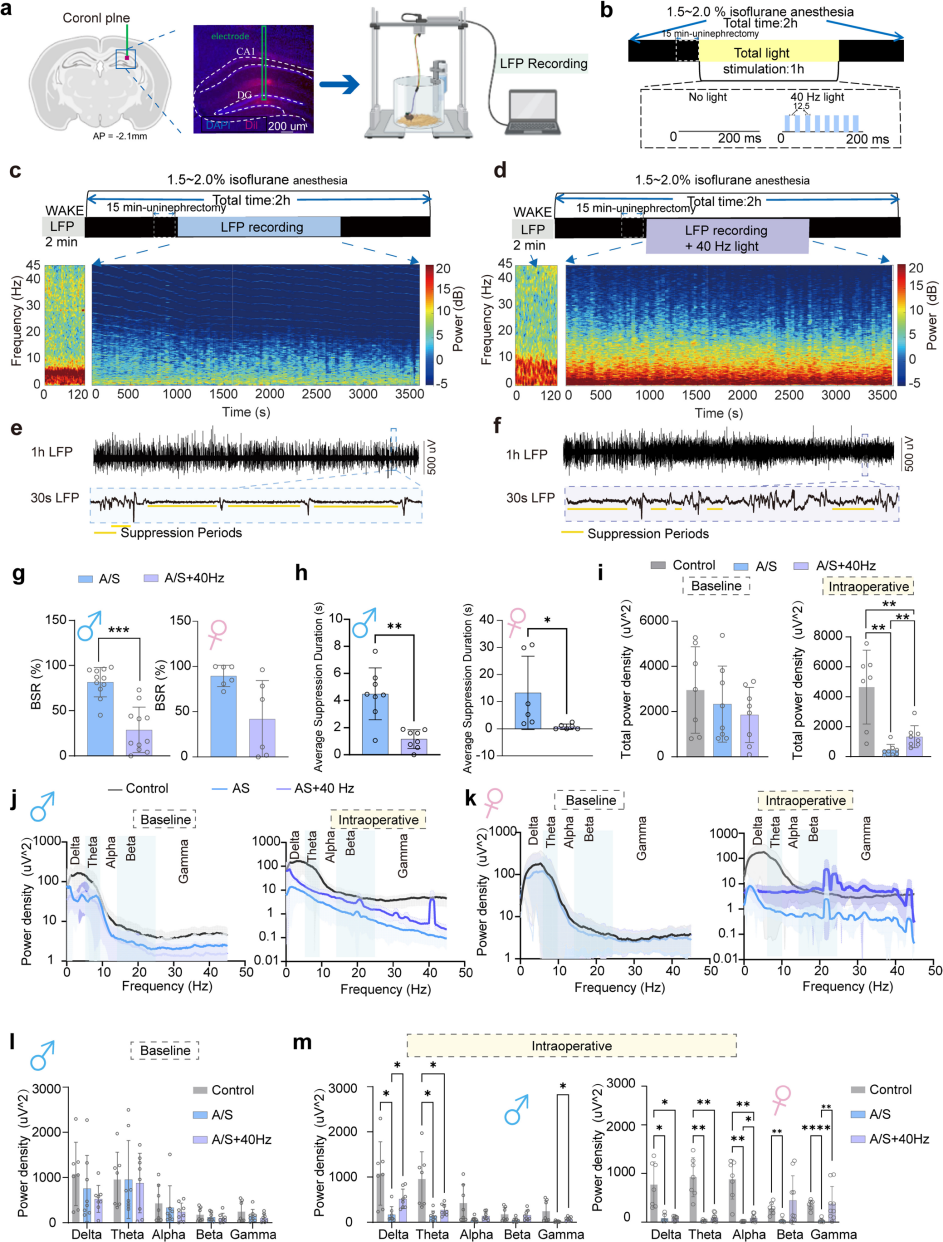
Administration of a 40 Hz visual light‐flicker intraoperatively minimizes local field potential suppression in the dentate gyrus of the hippocampus of mice. Male and female mice were randomly allocated to three groups, namely, the control group (control), anesthesia and surgery group (A/S), and anesthesia and surgery administered with 40 Hz visual light‐flicker group (A/S + 40 Hz). (a) Brain section schematic (created by biorender), image showing the track of the electrode probe and LFP recording schematic (b) Top: Experimental protocol for surgery with light flicker. Bottom: Parametric protocols for absence of light (left) and 40 Hz visual light flicker (right). (c) Top: Timeline of surgery and LFP recording. Bottom: Continuous A/S group preoperative and intraoperative LFP spectrograms. (d) Top: Timeline of surgery and LFP recording with 40 Hz visual light flicker. Bottom: Continuous A/S + 40 Hz group intraoperative and intraoperative LFP spectrograms. (e) Top: Continuous LFP Trace of A/S group for 1 h. Bottom: Representative 30‐s LFP Trace of A/S group. (f) Top: Continuous LFP Trace of A/S + 40 Hz group for 1 Hour. Bottom: Representative 30‐s LFP trace of A/S + 40 Hz group. (g) Statistical charts of the BSR of LFP in intraoperative recordings for male (left) and female (right) mice. BSR (%) = (Duration of Suppression Periods/Total Duration of EEG Recording) * 100. (h) Statistical charts of average suppression duration of LFP in intraoperative recordings for male (left) and female (right) mice. (i) Statistical charts of total power density at baseline (left) and during intraoperative periods (right) for male mice. (j) Quantification of power spectral density at baseline (left) and during intraoperative periods (right) for male mice. (k) Quantification of power spectral density at baseline (left) and during intraoperative periods (right) for female mice. Statistical charts of power density across different frequency bands at baseline for male mice. (m) Statistical charts of power density across different frequency bands during intraoperative periods for male mice (left) and female mice (right). Data are presented as mean ± SD with the presentation of data of each individual animal (male: *N* = 8; female: *N* = 8). Results were analyzed by one‐way or two‐way ANOVA and *t*‐test. *p < 0.05, **p < 0.01, ***p < 0.001, ****p < 0.0001. BSR, burst suppression ratio; DG, dentate gyrus; LFP, local field potential.

Figure 5a shows the surgical procedure, electrode locations, and setup. Baseline LFP was recorded during wakefulness prior to surgery and anesthesia. Following surgery, LFP recording continued under sustained anesthesia for 1 h in both the A/S and A/S + 40 Hz groups. Figure 5c,d show higher electrical power in the A/S + 40 Hz group across all frequencies. Raw data (Figure 5e,f) depict a typical 30‐s segment. BSR analysis assessed anesthetic suppression effects on hippocampal activity. Both the BSR and the average LFP suppression duration were significantly higher in the A/S group than in the A/S + 40 Hz group (Figure 5g,h, left and right for male and female mice respectively). We then compared the overall power of the field potentials. No significant differences were observed in the overall field potential power among the Control, A/S, and A/S + 40 Hz groups across frequency bands at baseline (Figure 5i for male and Figure S2d, left for female). However, during surgery, the field potential power in the A/S group was significantly lower than that in the A/S + 40 Hz group and the Control group (Figure 5i, right for male and Figure S2d, right for female). These findings suggest that 40 Hz visual light flicker during surgery reduces brain activity suppression induced by anesthesia and surgery. Spectral power analysis across frequencies showed no significant differences in overall field potential power among three groups under baseline conditions (male: Figure 5j, left and l; female: Figure 5k, left and Figure S2e). However, during surgery, the A/S + 40 Hz group exhibited a trend of increased power compared to the A/S group, with significant increases observed especially in gamma bands (Figure 5j, right and Figure 5m, left for male; Figure 5k, right and Figure 5m, right for male).

## 4 Discussion

This study examined the impact of 40 Hz visual light flicker on PND and its underlying neural mechanisms. The results demonstrated that 40 Hz light flicker for 1 h significantly improved mice performance in several cognitive tasks, including the Y‐maze, the novel object recognition, and conditioned fear tasks, compared to random, continuous, or no light exposure. These findings suggest that 40 Hz flicker holds potential for specifically improving cognitive impairment in PND mice. Previous studies have linked gamma frequency impairment in the hippocampus to cognitive dysfunction. Our results show that 40 Hz flicker not only reversed the decline in gamma frequency connectivity between the visual cortex and hippocampus but also alleviated gamma frequency suppression in a hippocampal region (high burst suppression rates and low power).

40 Hz sensory stimulation has shown significant effects in inducing gamma wave synchronization, improving cognitive function, and reducing amyloid pathology in Alzheimer's disease mouse models [12, 13, 14, 15], although the overall efficacy remains a subject of ongoing research [11]. Therefore, when setting parameters for various types of light stimulation, we chose 40 Hz as the regular frequency. Additionally, we implemented continuous light and light stimuli with the same duration but random flashes to control for the effects of visual stimulus intensity and flicker. Our results indicated that 40 Hz regular light stimulation produced superior experimental outcomes, offering valuable insights for the future clinical application of light‐flicker stimulation in improving PND.

This experiment aimed to examine the effects of intraoperative visual light stimulation on PND. Previous studies on 40 Hz light stimulation have primarily focused on AD models, often involving awake mice or patients with prolonged sensory stimulation [3, 12, 13, 43]. These interventions were conducted in awake subjects, which differ from our approach of applying light‐flicker stimulation during surgery. The rationale for applying 40 Hz stimulation during surgery (intraoperatively) is twofold, based on the acute pathophysiology of PND. First, it aims to intervene at the critical moment of pathogenesis. Unlike Alzheimer's disease, which is a slowly progressive neurodegenerative disorder, PND typically manifests soon after surgery. Both surgical trauma [44, 45, 46] and exposure to general anesthesia [44, 45] are known to initiate key pathological processes—such as neuroinflammation and neuronal dysfunction—that underlie PND onset. Second, intraoperative stimulation represents a preventive strategy. By targeting these mechanisms (e.g., modulating neural synchrony and potentially mitigating inflammation) as they are initiated, we hypothesize that it may prevent or attenuate the subsequent cascade leading to cognitive decline, rather than attempting to reverse established pathology postoperatively. However, the potential for post‐operative light stimulation to improve cognitive function requires further investigation.

Although anesthesia‐induced local field potential suppression generally affects memory‐related brain regions (e.g., the hippocampus), previous studies have demonstrated that intraoperative field potential suppression and impaired functional connectivity are significant risk factors for PND [3]. Our study also revealed that intraoperative light‐flicker stimulation significantly enhances field potential activity in the visual‐hippocampal circuit, as evidenced by increased gamma power within each region (indicating enhanced local synchronization) and restored interregional functional connectivity between them. However, these stimulatory effects—enhanced local gamma synchronization and restored long‐range connectivity—are transient and not the primary contributors to PND [47, 48]. The pathological mechanisms underlying PND are more closely associated with the inflammatory response induced by surgical procedures during the intraoperative period. Deep anesthesia may exacerbate the intraoperative inflammatory response, with pro‐inflammatory factors (e.g., IL‐1β, TNF‐α) crossing the blood–brain barrier and entering the central nervous system, thereby activating microglia and inducing neuroinflammation, which subsequently exacerbates cognitive dysfunction [49, 50]. Therefore, enhanced visual light stimulation may alleviate anesthesia‐induced field potential suppression, improves regional brain connectivity, which collectively may contribute to facilitating the recovery of postoperative cognitive function.

In our study, merely increasing sensory input (such as continuous light and random light) did not improve postoperative cognitive function, whereas 40 Hz light‐flicker stimulation significantly enhanced cognitive performance. 40 Hz light‐flicker stimulation has been demonstrated to enhance cognitive function through multiple mechanisms. These mechanisms include enhancing gamma synchronization within neural circuits, modulating the phase‐locking of GABAergic neurons, regulating the activity of non‐neuronal cells (such as microglia and astrocytes), influencing gene expression, offering neuroprotective effects (e.g., reducing amyloid plaques and tau phosphorylation) and improving cerebral blood flow and vascular function [3, 12, 13, 14, 15]. Cross‐brain region studies have suggested that gamma frequency coherence can alleviate neuronal degeneration in the early stages of neurodegenerative diseases, improve synaptic function, and consequently mitigate cognitive dysfunction [14]. Intraoperatively, we observed a significant decline in gamma functional connectivity between the visual cortex and hippocampus, which may contribute to the decrease in hippocampal gamma power, potentially representing a critical mechanism of intraoperative cognitive dysfunction. However, light stimulation effectively reversed this change and by restoring the gamma‐frequency coherence specifically within the visual‐hippocampal circuit, it may represent a key mechanism by which 40 Hz light‐flicker stimulation improves cognitive function. Despite the observable behavioral improvement, the specific molecular mechanisms underlying this effect still require further investigation.

We found that intraoperative 40 Hz flicker stimulation effectively improves postoperative cognitive function in both sexes, indicating a robust, sex‐independent benefit at the behavioral level. Although spectral analysis revealed differences in the breadth of frequency bands affected, a significant enhancement in gamma‐band coherence between V1 and DG was a consistent and prominent finding in both male and female mice. These findings suggest that males and females may engage distinct frequency‐specific pathways to achieve similar cognitive recovery. Electroencephalographic (EEG) studies revealed that this stimulation markedly reversed the decline in gamma‐band functional connectivity. Potential causes of sex differences in cognitive function include: (a) sex hormone regulation [51, 52, 53]. (b) X‐chromosome dosage effects [54] (c) Sex‐specific genetic pathways (female: immune/inflammatory [55]; male: cardiovascular [56]). (d) Sociocultural factors (education, gender roles) [57, 58]. Crucially, we found that the stimulation significantly reversed anesthesia‐induced declines in gamma‐band functional connectivity (as measured by EEG), a key neural mechanism impaired postoperatively that is independent of sex [59]. By effectively targeting this common, sex‐independent neural deficit (suppression of functional connectivity), the 40 Hz light stimulation achieves equivalent cognitive benefits in both sexes.

This study has notable limitations requiring attention. First, while 40 Hz visual stimulation shows therapeutic promise in ameliorating PND in aged mice, optimal stimulation parameters (e.g., intensity, wavelength, duration) warrant systematic investigation. Second, longitudinal studies are crucial to ascertain the intervention's sustained efficacy and temporal effects. Third, it is important to note that this study utilized a combined anesthesia/surgery model. Future studies incorporating an anesthesia‐only control group will be essential to delineate the specific contributions of anesthetic exposure versus surgical trauma to the observed neurocognitive impairments and the protective mechanisms of sensory stimulation. Fourth, heterogeneous responses across cognitive impairment subtypes highlight the necessity for personalized treatment protocols and parameter optimization. Fifth, the mechanistic basis remains incompletely elucidated—though photosensitive retinal ganglion cell modulation appears pivotal, detailed circuit‐level mechanisms demand further exploration. Continued investigation is imperative to optimize this approach for clinical translation. Lastly, the female and male cohorts were run in separate experimental batches, precluding us from pooling the data for a consolidated analyses of potential sex differences. Future studies should co‐test sexes within the same experimental batches to explicitly address this variable.

In summary, the results demonstrate that 40 Hz visual light‐flicker stimulation, rather than other types of light flicker, can improve postoperative cognitive function by attenuating PND. This effect may occur through the activation of the visual cortex, which reverses the decline in gamma frequency functional connectivity between the visual cortex and hippocampus, subsequently increasing gamma oscillation power in the hippocampal region. This suggests that stimulation enhances gamma frequency coherence and restores functional connectivity within the visual‐hippocampal circuit, thereby improving cognitive function. These findings provide insights into PND prevention and the neural mechanisms underlying 40 Hz‐induced cognitive benefits.

## Funding

This work was supported by the National Natural Science Foundation of China [grant numbers 82201341, 82371281, 82271290]; Natural Science Foundation of Sichuan Province [grant numbers 2024NSFSC1635]; Sichuan Provincial Department of Science and Technology [grant numbers 2023NSFSC0676, 2023ZYD0168].

## Ethics Statement

The experimental protocol of this study was conducted in compliance with the Animal Research Reporting of In Vivo Experiments (ARRIVE) guidelines and was approved by the Animal Ethics Committee of West China Hospital, Sichuan University (Approval No. 20231109005; issued November 9, 2023).

## Conflicts of Interest

The authors declare no conflicts of interest.

## Supporting information


**Figure S1:** Anesthesia, surgery, and 40 Hz visual light flicker did not significantly impair the locomotor abilities of the mice.
**Figure S2:** Field potential recordings in female mice.

## Data Availability

The data that support the findings of this study are available from the corresponding author upon reasonable request.
