# Supplementary material for: Intraoperative 40 Hz Visual Light Flicker Attenuates Anesthesia/Surgery‐Induced Cognitive Impairments in Elderly Mice With Enhanced Cortical‐Hippocampal Coherence
Source: CNS Neurosci Ther. 2026 Mar 7;32(3):e70809. doi: 10.1002/cns.70809 (PMC12967628; doi:10.1002/cns.70809)
Supplement: Supplementary file 1 — Figure S1: Anesthesia, surgery, and 40 Hz visual light flicker did not significantly impair the locomotor abilities of the mice. Figure S2: Field potential recordings in female mice. [file CNS-32-e70809-s001.docx]

**
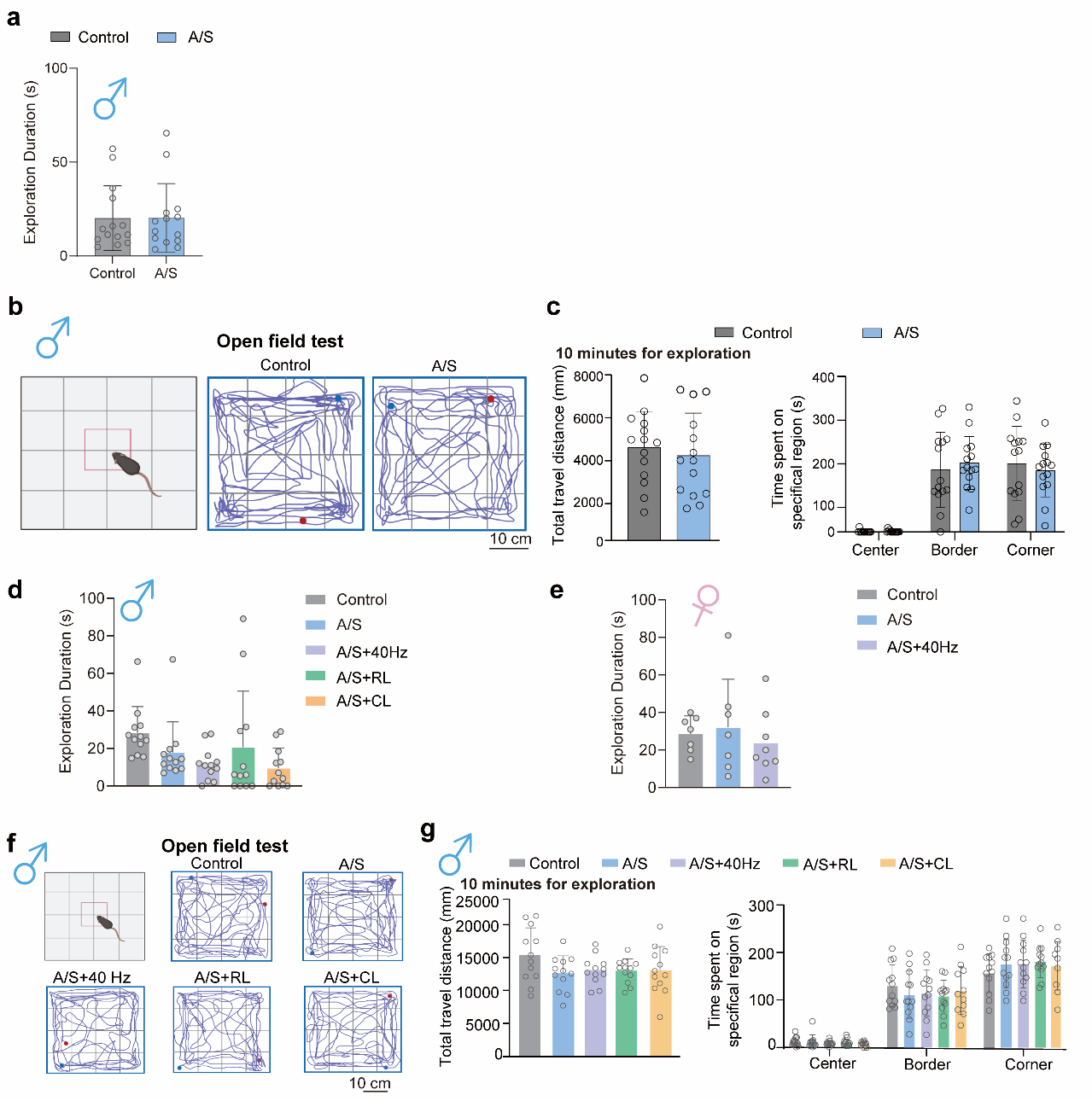
**

**Fig S1.** **Anesthesia, surgery, and 40 Hz visual light flicker did not significantly impair the locomotor abilities of the mice.**

Mice were randomly assigned to five groups: the control group (Control), the anesthesia and surgery group (A/S), the anesthesia and surgery group with 40 Hz visual light flickering (A/S + 40 Hz), the anesthesia and surgery group with random frequency light flickering (A/S + RL), and the anesthesia and surgery group with continuous light flickering (A/S + CL).

(a) Statistical chart of total exploration duration for male mice in the Novel Object Recognition test.

(b) Description of the Open Field Test methodology (left) and representative movement trace diagrams in the Open Field Test; the red and blue dots indicate the start and end points of the trajectory, respectively.

(c) Statistical charts showing total traveled distance (left) and time spent in specific regions (right) in the Open Field Test.

(d) Statistical chart of total exploration duration for male mice in the Novel Object Recognition test.

(e) Statistical chart of total exploration duration for female mice in the Novel Object Recognition test.

(f) Description of the Open Field Test methodology and representative movement trace diagrams in the Open Field Test; the red and blue dots indicate the start and end points of the trajectory, respectively.
(g) Statistical charts showing total traveled distance (left) and time spent in specific regions (right) in the Open Field Test.

Data (n = 14 for males, n = 8 for females) are presented as mean ± S.D., with individual data points shown. Results were analyzed by one-way ANOVA.

**
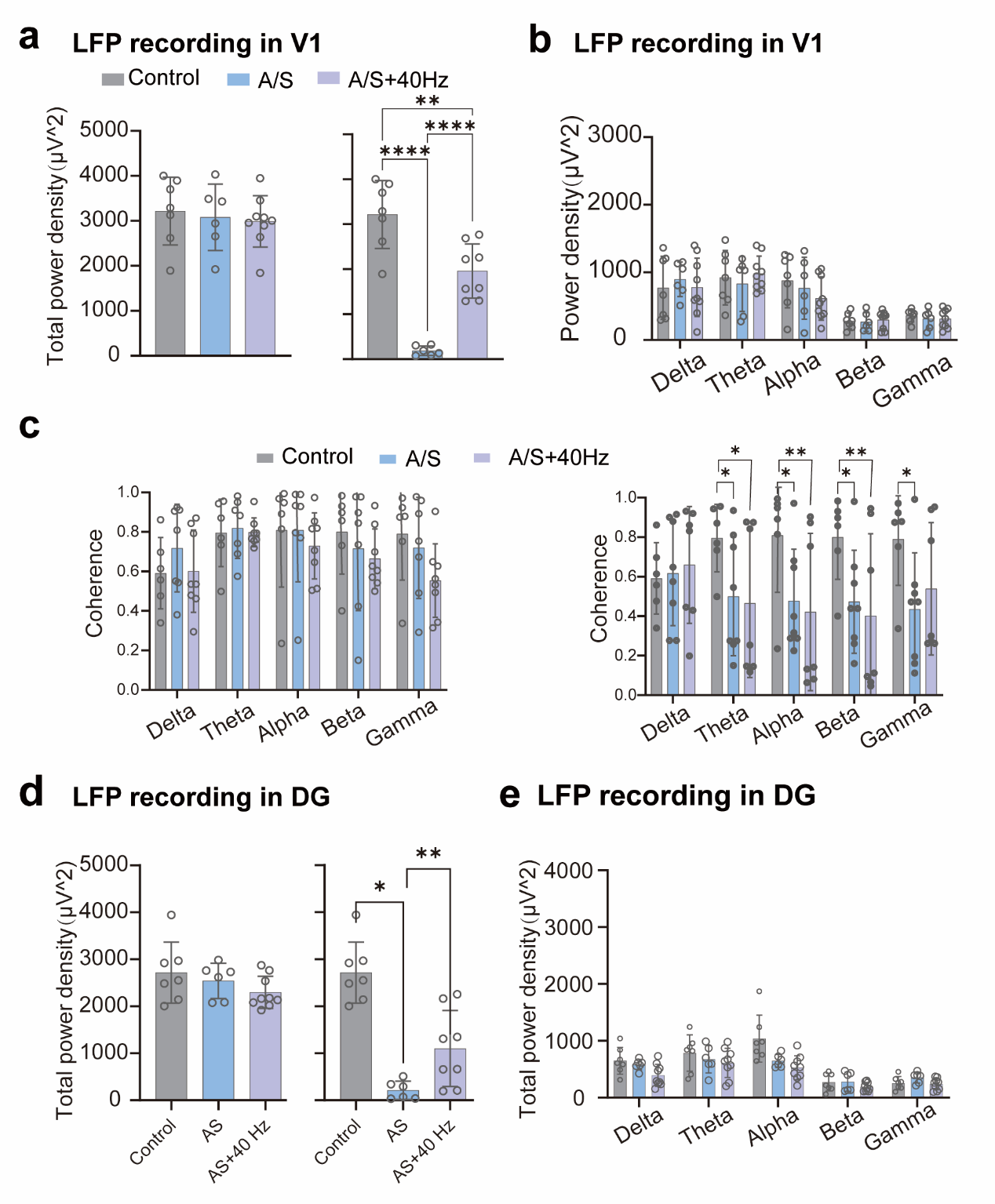
**

**Fig S2.** **Field potential recordings in female mice**

Mice were randomly assigned to five groups: the control group (Control), the anesthesia and surgery group (A/S), the anesthesia and surgery group with 40 Hz visual light flickering (A/S + 40 Hz).

(a) Statistical charts of total power density in V1 at baseline (left) and during intraoperative periods (right) for female mice.

(b) Statistical charts of power density across different frequency bands in V1 at baseline for female mice.

(c) Statistical charts of coherence at baseline (left) and during the intraoperative period (right) for male mice across the Control, A/S and A/S + 40 Hz groups.

(d) Statistical charts of total power density in DG at baseline (left) and during intraoperative periods (right) for female mice.

(d) Statistical charts of power density across different frequency bands in DG at baseline for female mice.

Data are presented as mean ± SD with the presentation of data of each individual animal (n = 8). Results were analyzed by one-way or two-way measures ANOVA and t-test. *, P<0.05, **, P<0.01, ****, P< 0.0001. V1, primary visual cortex**;** LFP, local field potential; A/S, anesthesia and surgery group; DG, dentate gyrus.
